# Consistency Analysis of Assessment Boards in University Entrance Examinations in Spain

**DOI:** 10.3390/ejihpe15060102

**Published:** 2025-06-05

**Authors:** Alejandro Veas, José-Antonio López-Pina

**Affiliations:** 1 Department of Developmental and Educational Psychology, University of Murcia, 30100 Murcia, Spain; 2 Department of Basic Psychology and Methodology, University of Murcia, 30100 Murcia, Spain; jlpina@um.es

**Keywords:** comparability approach, correction criteria, higher education, Many-Facet Rasch Model, raters, university entrance examinations

## Abstract

University entrance examinations (EBAU in Spanish) are a nationwide program for assessing student achievement levels in Spain and determining access to public undergraduate degrees. Considering the need to measure the progress of rater performance, this study analyzes rating data from the June 2018 exam sitting in the Valencian Community, Spain. A total of 54 assessment boards and 3000 students from five public universities were observed. The Many-Facet Rasch Model (MFRM) was used as an extension of the one-parameter Rasch measurement model. All facets involved in analyses (examination board severity, subject difficulty, and group performance) were located on a common underlying linear scale. The results showed large inconsistencies in the rating process, with differences in the severity levels of many subjects both within and between universities. This study may serve as a starting point for a reflective debate on the need to apply better criteria for ensuring the comparability of examination standards in Spain.

## 1. Introduction

Assessment processes are an essential tool for training and measuring the impact of educational systems. While the purpose of the assessment may vary, many countries have focused on determining how the qualitative and quantitative analyses of standards impact the quality and accuracy of relevant national examinations (Baird et al., 2000; Blyth, 2014; Coe, 2007; He et al., 2018; Meadows et al., 2025; Stringer, 2012).

In Spain, one of the main assessment instruments is the “Baccalaureate Examinations for University Entrance” (EBAU). These examinations are designed to assess the academic readiness of students who wish to enter higher education. To that end, the EBAU allows students to demonstrate their knowledge of and skills in various subjects after completing two Baccalaureate courses.

There have been several attempts in Spain to analyze the difficulty or inter-subject comparability of examinations (Cuxart-Jardí, 1996, 2000; Cuxart-Jardí & Longford, 1998). However, the political and administrative regulation of the country produces difficulties in statistically ensuring equal examinations. Each Spanish Region is autonomous and can design specific tests, following minimal regulation standards according to Spanish law (Order PCI/12/2019). In addition, the analysis of difficulties among examinations has been linked to possible rater effects (European Commission/EACEA/Eurydice, 2009). In this regard, Eckes (2009) proposed two main sources of error that affect objectivity in assessment: distal and proximal factors. Distal factors refer to aspects such as student characteristics and assessment context. Some variations are related to gender and age. Proximal factors, however, refer to the construct being measured, such as the structure of score categories and grading criteria.

In high-stakes assessments, multiple studies reported relevant results regarding rater effects and the impact of rubrics and standard criteria. Jonsson and Svingby (2007) stated that analytical rather than holistic rubrics are preferable in this type of assessment. In a recent study, Holmes et al. (2018) detected a strong correlation between expected difficulty using the comparative judgment approach and actual difficulty derived from a Rasch analysis of the GCSE mathematics examination. Thus, considering the relevant research on this topic, comparability measures would be more reliable indicators if judgment criteria were also explored. This study aims to explore the consistency rating of boards in Spanish university entrance examinations.

### 1.1. Antecedents

Prior studies have mainly focused on developing predictive explanatory models of achievement in Spain, including in the EBAU context. During the 1980s and 1990s, inequality access was explored through analyzing selection criteria (Cuxart-Jardí et al., 1997; Goberna et al., 1987). These studies confirmed the importance of considering the heterogeneity of measures and their impact on both university enrolment and objective selection criteria. Differences could be found in personal variables, chosen scientific degree, and the relationships between the predictive power of subjects and undergraduate degrees analyzed (Cuxart-Jardí, 2000; Rodríguez-Menéndez et al., 2014; Ruiz et al., 2011). In addition, quasi-experimental studies were conducted to analyze the differences between raters and boards in both total and partial scores of examinations. For instance, Escudero-Escorza and Bueno-García (1994) studied the scoring patterns of two additional boards that assessed eight examinations. They detected insignificant and minimal differences in the proportion of students who pass the entire examination process and concluded that the correction patterns were quite similar among the boards. In another quasi-experimental study, Watts and García-Carbonell (1999) selected two random boards of four raters to detect possible differences in assessment patterns in the English examination, depending on the use of holistic or focused-holistic correction criteria. The results confirmed higher levels of inter-rater reliability and accuracy when using focused-holistic criteria.

Beyond variable comparison, statistical procedures in EBAU were also considered. Gaviria (2005) explored various statistical techniques (classical, ordinary least squares, multilevel, average equalization, and standard deviation methods) to analyze the equivalence of baccalaureate grades with EBAU exam grades, with the latter being an anchoring point as it is a standardized test for all students. The results showed that non-classical methods produce better results than the classical weighting method and make for a fairer student selection process. To include modern statistical methods, such as those applied in the international context, Veas et al. (2020) investigated the construct comparability approach (Coe, 2008, 2010) through the Rasch model to compare both fit and difficulty levels among subjects from the 2018 EBAU exam sitting in the Valencian Community. The results showed adequate difficulty levels across the student ability continuum, though a lack of discrimination between high and low ability students was detected. Important conclusions are addressed, such as the influence of the difficulty of the subject selected by students in the examination process and the need to review score categories across subjects to obtain more reliable scores.

In the international context, the possible influence of raters has also been explored (Bejar, 2012; Congdon & McQueen, 2000; European Commission/EACEA/Eurydice, 2009). For assessment procedures implemented at various educational levels, most studies reported rater effects on the use of rubrics and assessment criteria. Jonsson and Svingby (2007) claimed that analytic rather than holistic rubrics are better in this type of assessment. Moreover, Holmes et al. (2018) showed that the pairwise comparison method had high levels of predictions of difficulty in mathematics tests, confirming the efficacy of methods such as the Rasch model. In this vein, the scientific literature confirms the need to include rater effects in statistical modeling to ensure the reliability and consistency of cut-off scores (Benton & Elliot, 2016). The use of predictions between cohorts of students examined in different years to ensure reliable standards in official examinations is most common in the UK (Benton & Sutch, 2014; Leckie & Baird, 2011).

### 1.2. The Present Study

Assessment presents a complex structure in the design process at all educational levels. According to Mislevy et al. (2003), the real challenge of all systems is to provide a framework supported by cognitive psychology, measurement models, information technology, and learning in disciplines. However, there are several differences in standard methods applied in various countries and even in inferences or arguments used to confirm the assessment structure used (Baird et al., 2024; Ehren, 2023). The present study tries to bridge this gap by extending advanced measurement models into EBAU, one of the main types of examinations in Spain. Indeed, analyses of raters or examination boards have received some attention in the last few decades; however, in spite of their importance for measuring grading quality, these studies used small samples of examinations and boards in local areas. Evidence shows that the level of demand and difficulty among different exam boards should be controlled by statistical evidence gathered from a range of sources (Baird et al., 2000; Robinson, 2007). Therefore, this study has two main aims: (a) to test the severity and leniency levels of examination boards in an official EBAU exam sitting in a region of Spain; (b) to explore severity and leniency levels applied to each subject for each participant university.

Despite having no previous studies to determine clear directional hypotheses, previous difficulty-level analyses with the Rating Scale Rasch Model (RSRM, Andrich, 1978) showed adequate difficulty levels in the analysis of subjects. It is, thus, possible to obtain positive results, considering the specific and public assessment rubrics used by examination boards.

This study is structured to present a detailed analysis of assessment consistency in the EBAU system. The subsequent section outlines the research methodology, which employs the Many-Facet Rasch model to examine scoring variances across multiple examination boards, addressing potential rater effects and the complexities of the evaluation process. The findings section then conveys the results regarding the score disparities, supporting the need for standardized assessment practices to promote fairness. Finally, the discussion section reflects on the implications of these findings for the educational policy, with recommendations aimed at fostering inter-subject comparability and enhancing transparency in the assessment system.

## 2. Materials and Methods

### 2.1. Participants

We used a subsample from a large-scale study based on a national research project, which aimed to explore the measurement properties of EBAU examinations in Spain. In our study, 3000 participants were randomly selected from 19,690 students in the June 2018 exam sitting, with an equal proportion of students recruited from three provinces (Alicante, Valencia, and Castellón), for estimating parameters; this was the only region from which the rating score system could be analyzed. A total of 54 examination boards were used for data analyses. Individual raters could not be considered because, to ensure anonymization, the local government only provided data from general boards. Consequently, only the total number of boards was computed, together with the total number of students assigned, the province, and the university. The frequency distributions of boards and students can be seen in Table 1 and Table 2, respectively.

### 2.2. Instruments

This study used a selected group of EBAU examinations administered in the Valencian Community, which was the same for all three provinces (Alicante, Valencia, and Castellon). The selection criterion was a minimum of 600 students tested per academic subject. This was used to ensure the greater precision of the estimated parameters (He et al., 2018). The academic subjects selected were Castilian Language and Literature, History of Spain, English Language, Mathematics II, Applied Mathematics for Social Sciences II, and Valencian Language.

### 2.3. Procedure

Permission was first obtained from the University Regulation Service (SIIU), the formal institution belonging to the Autonomous Government of Valencia, which provided grades from all students enrolled. For the present research, data from June 2018 were taken for analysis, considering 54 examination boards across the three provinces. Table 2 shows the number of students enrolled per examination board, as well as the provinces and cities where examinations were held. Data were totally anonymized and registered at SIIU, and no previous informed consent was needed. Panel boards are coordinated by an EBAU commission, which comprises representatives from the Department of Education (e.g., the chief of the educational assessment section), high school directors, educational inspectors, and both university and high school professors.

Panel boards have two main functions: (1) to create the questions for the specific subject’s official EBAU call and (2) to elaborate and justify the standard correction criteria and the marking score distributed for each question. Boards are assigned to a concrete local area and a university.

### 2.4. Data Analysis

For the present study, the Many-Facet Rasch Model (MFRM, Linacre, 1993) was used as an extension of the Rasch measurement model (Rasch, 1960). The MFRM has important advantages over classical data analysis. First, all facets involved in analysis (examination board severity, subject difficulty, and group performance) are located on a common underlying linear scale. This results in a measure that can be subjected to traditional statistical analysis, while allowing for unambiguous interpretation of group performance as it relates to board severity and subject (or item) difficulty. Second, calibration does not depend on the sample used. This means that the Rasch technique removes the influence of sampling variability from its measures so that valid generalizations can be made. In our specific EBAU analysis, this was important for dealing with missing data, as not all students were enrolled in the same subjects, and not all boards rated the same students. Third, fit procedures exist to derive unexpected response patterns useful for evaluating the extent to which individual groups, tasks, or raters (in our case, examination boards) behave in ways inconsistent with the measurement model (Engelhard, 1992).

The MFRM allows users to create a single interval of scores relevant to both the difficulty of subjects and the ability of the persons tested. These scores are usually reported in logits due to their easy arithmetic properties in terms of interpretations. In this study, the MFRM was deemed a more effective statistical procedure since multiple examination boards assess different groups of students based on standard rubrics for each subject. Moreover, the MFRM does not require assumptions about sampling or the normality of distributions; thus, it is useful for performance assessment with different subject structures at any educational level, even higher education. The formulation of the facet model to perform the analysis in this study was conducted as follows:

(1) $$log\frac{P}{1-P}=rater+item+step+rater\times item$$

where *rater* is the university, *item* is the subject, and *step* is the grade obtained in the subject. The parameter estimation was performed using the marginal maximum likelihood method.

For this analysis, the adequacy of scores was checked with values of examination boards’ mean square residuals (MNSQs), following criteria from Eckes (2005) and Wolfe and McVay (2012). Estimators of the severity of each board, together with estimating the mean score severity for each subject, were used to determine the adequacy of scores.

Unweighted mean square and weighted mean square statistics were used to check rater severity levels from a Rasch measurement perspective. Weighted mean square statistics give more importance to boards aligned with the ability level of subjects. More weight is provided to these subjects as they can carry more information about board ability. The weighted mean square was defined as follows:

(2) $$WMS=\frac{\sum {\left[U_{ij}-P_{ij}\left(\theta \right)\right]}^{2}}{\sum w_{ij}}$$

where $U_{ij}$ is the rating obtained for the student, $P_{ij}\left(\theta \right)$ is the probability of obtaining that rating based on the proposed measurement model, and $w_{ij}=P_{ij}\left(\theta \right)\left[1-P_{ij}\left(\theta \right)\right]$.

In contrast, unweighted mean square statistics give more importance to boards that rated subjects positively far above their ability level (Bond & Fox, 2007). The unweighted mean square was defined as follows:

(3) $$UMS=\frac{1}{N}\sum \frac{{\left[U_{ij}-P_{ij}\left(\theta \right)\right]}^{2}}{w_{ij}}$$

where *N* is the sample size, and all other terms are interpreted as in *WMS*.

Values of unweighted and weighted mean squares (MNSQs) can range from 0 to positive infinity. Values below 1 indicate a better fit than expected using the model, whereas values above 1 indicate a poorer fit than expected using the model. In this case, the appropriate range for fit statistics was 0.5–1.5 (Wright & Linacre, 1994). These fit statistics could be approximately normalized using the Wilson–Hilferty transformation with *t*-values. The Wilson–Hilferty *t*-transformation has the following form:

(4) $$t=\left[\left({MS}^{1/3}-1\right)\left(\frac{3}{\sigma }\right)\right]+\left[\frac{\sigma }{3}\right]$$

where *MS* is the mean square weighted or unweighted value, and *σ* is the standard deviation of the mean square. This transformation follows a normal distribution, with a mean of 0 and a standard deviation of 1, where values within a range from −2 to +2 are considered appropriate, although this statistic may not follow a normal distribution in high sample sizes. Conquest version 2.0 (Wu et al., 2007) was used to conduct analyses in this study.

## 3. Results

Rater effects were assessed by examining the severity level of each board (Table 3). All estimations had adequate unweighted and weighted fit indices, indicating the good fit of the data to the model. Important heterogeneity levels were detected among boards within and between universities. The most severe was board 5-3, with 0.725 logits, followed by board 4-8, with 0.658 logits.

Table 4 shows the total severity levels of boards for each subject. Applied Mathematics for Social Sciences (MCS) had the highest severity score, with 0.611 logits, followed by Mathematics (MAT), with −0.015; Castilian Language and Literature (CAS), with −0.099; English Language (ENG), with −0.675; Valencian Language and Literature (VAL), with -0.736; and History of Spain (HES), with −1.070 logits. Students enrolled in experimental sciences have higher severity scores compared to those enrolled in humanities or social sciences.

**Figure 1:**
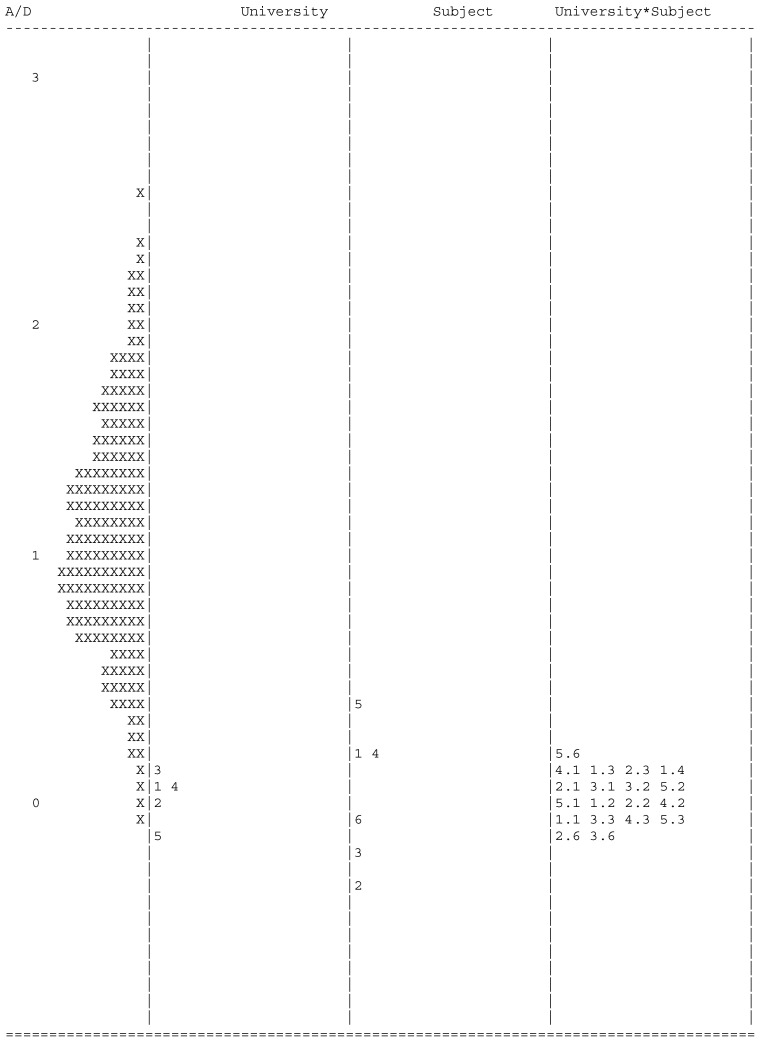
Map of latent distributions and response model parameter estimates. *Note.* Each ‘X’ represents 15.5 cases. A/D = Ability/Difficulty parameters; Second column: 1 = University of Alicante; 2 = University Miguel Hernández; 3 = University of Valencia; 4 = Polytechnic School of Valencia; 5 = University Jaume I. Third Column: 1 = Spanish Language and Literature; 2 = History of Spain; 3 = English Language; 4 = Mathematics II; 5 = Mathematics Applied to Social Sciences; 6 = Valencian Language.

These differences are more extreme when comparing the severity mean scores of subjects across universities (see Table 5 and Figure 1). An example is the Valencian Language, as the severity mean score was 0.022 for the University of Valencia and 0.734 for the University Miguel-Hernández (a difference of 0.712 logits). There are no subjects with a similar pattern of severity mean scores across the five universities. Thus, students clearly benefit or are punished depending on the board to which they have been assigned and even the province where they are located. The Wright map confirms the visualization of these differences in the latent scale when considering the interaction of university and examinations (see Figure 1). This figure shows the distribution of students on the left axis of the figure from less ability (bottom) to more ability (top). The results of the estimated ability parameters suggest a normal distribution. Since, in the many-facet model, ability and difficulty parameters are on the same continuum, universities, subjects, and boards appear on adjacent axes according to the corresponding difficulty. The estimates of the difficulty parameters at the bottom of the figure indicate less university or board difficulty, while those at the top of the figure indicate more university or board difficulty.

## 4. Discussion

The present study explores the adequacy of the consistency scoring system of the examination boards of EBAU, one of the main examinations in Spain. Identifying and describing a consistent framework for monitoring rater performance is essential for analyzing the use of standards (Myford & Wolfe, 2009) and for ensuring student equity.

The results suggest a lack of homogeneity in the rating score system, even with the clear published standards that should be applied by all examination boards. According to Engelhard and Wind (2018), these results affect two main principles applied to rater-mediated assessment: the rater-invariant measurement of persons (*the measurement of persons must be independent of the particular raters that happen to be used in assessment*) and the invariant locations of raters (*locations of raters must be independent of the particular persons, cues, and rating scales used in assessment*). Therefore, students can benefit or be punished depending on their location or subject. This might also affect subjects selected by students, based on their future academic choice (knowledge field of the undergraduate degree).

Given the nature of EBAU, the diversity of the format of rating used across subjects may have some impact on the scoring consistency of examination boards. Some subjects, such as English Language, have multiple-choice and short-answer items, and they are given a more objective correction system where success or failure has a direct score. However, in others, such as History of Spain, students must write an elaborate comment on a text or respond to broader questions. These types of written compositions are related to expanded rubrics, where the range of ratings can determine more differences among raters to accurately measure the construct. In this regard, we believe that Spanish national and local educational institutions should provide more consistent guidance for high-stakes examinations. It would be necessary to collect national data from individual raters to obtain more reliable results concerning rating accuracy. Moreover, analyzing rating performance over time would provide relevant information, for instance, to choose strategies to detect and measure changes in rater behavior or to select a frame of reference to depict drift (Myford & Wolfe, 2009).

A further important issue is student equality regarding university access in Spain. The Spanish autonomous communities create their own exams with minimum standards approved by the Spanish government; nevertheless, students may apply to any public university nationwide regardless of the region they belong to. A lack of comparative analyses of the rating score tendency of the autonomous communities constitutes a serious threat to the rating process. The present study may serve as a starting point to determine future replications for ensuring consistent and generalized rating strategies. Regarding examination entry policy, as students may receive a different impact on their achievement according to rating severity, the number and type of subjects chosen may vary across autonomous communities. If so, this strategy overlaps with the need to choose more exams to achieve a better access grade, affecting current achievement levels (Newton, 1997).

Given the importance of an accurate grading system in the EBAUs and the results obtained, the authors of this manuscript propose some organizational and methodological strategies that can be effective in the future. In the first place, it would be necessary to systematically determine the same analytical level of correction criteria across examinations, as it seems to help ensure grading objectivity and reduce error deviations (Watts & García-Carbonell, 1999). As this procedure has been more studied in the field of English Language (Barkaoui, 2010; Yan & Chuang, 2023), it can be extended to other examinations that involve replies to similarly formatted questions that balance the holistic and analytic approaches (Tomas et al., 2019). Secondly, it is necessary to assess the reliability of the grading process. To this end, the leniency or severity levels of boards can be examined across consecutive years to check standard stability over time. This measure would lead to a more realistic comparison of regions or examination districts.

Finally, it is important to mention some limitations. First, only data from one region of Spain (Valencia) were used to analyze rater effects due to formal restrictions or difficulties in accessing data in other regions. Additionally, only numbered boards were registered, and not individual raters. Autonomous regions in Spain do not have a mandatory protocol to register individual raters, as, normally, this type of data is not required to be collected from the University Information System. We would like this study to be a starting point for checking and elaborating new data collection protocols for all the regions of the country to ensure the general equivalence of standards among exam boards regarding achievement criteria. Additionally, this study does not include data from different cohorts across time. The research literature has shown that the behavior of individual raters may change over time, and that DRIFT-differential rater functioning (Wolfe & Moulder, 1999) is ultimately a necessary measurement process for detecting differential accuracy and scale category use (Myford & Wolfe, 2009; Wolfe et al., 2001). We hope that this type of analysis can also be implemented in the future with extended samples and individual raters from different regions of Spain.

In conclusion, this study serves as a critical foundation for contemplating the inherent shortcomings of the EBAU system, particularly concerning equitable access. Political and social debate have discussed the negative consequences of an unequal selection of students in tertiary education (Faura-Martínez et al., 2022; Lamprianou, 2009). Therefore, it is strongly necessary that national policy prioritizes coordinated efforts among educational institutions and local authorities to develop effective strategies aimed at enhancing inter-subject comparability in assessments.

A comprehensive exploration and comparison of various assessment methodologies is necessary to foster greater uniformity in the difficulty levels of examinations, especially in the area of assessment rater effects (Guo & Wind, 2021; Zabala-Delgado, 2021). Such an approach would ensure that the implementation of the unique district procedure remains transparent, equitable, and ultimately conducive to a fairer selection process according to the promotion of social justice in the international context (Volante et al., 2024). Moreover, effective educational reform is contingent upon active participation by stakeholders in the policy development process, ensuring that reforms are responsive to actual educational needs and challenges (Poskitt, 2023). National policy reform would acknowledge the urgent need for systemic changes that address these critical issues, thereby reaffirming the commitment to equity and excellence in education across all autonomous communities in Spain.

## Figures and Tables

**Table 1:** Characteristics of the university entrance examination boards.

Province	University	Number of Boards	Number of Students	Number of Students Included in This Study
Alicante	(1)	11	3306	522
	(2)	10	3403	509
Valencia	(3)	15	5848	883
	(4)	12	4885	752
Castellon	(5)	6	2248	334
Total		54	19,690	3000

*Note.* (1) University of Alicante, (2) University Miguel-Hernandez, (3) University of Valencia, (4) Polytechnic University of Valencia, and (5) University Jaume I.

**Table 2:** Frequency and percent of participants in the subjects for each university.

Subject	U. Alicante	U. Miguel-Hernandez	U. Valencia	Polytechnic School of Valencia	U. Jaume I
	Freq (%)	Freq (%)	Freq (%)	Freq (%)	Freq (%)
CAS	470 (17.5)	469 (17.5)	796 (29.7)	637 (23.8)	308 (11.5)
HES	470 (17.5)	470 (17.5)	797 (29.7)	638 (23.8)	308 (11.5)
ING	463 (17.1)	460 (17.5)	778 (29.7)	627 (23.9)	294 (11.2)
MAT	250 (17.8)	234 (16.7)	395 (28.1)	352 (25.1)	173 (12.3)
MCS	170 (16.1)	170 (16.1)	350 (33.1)	243 (22.9)	126 (11.9)
VAL	409 (17.2)	320 (13.5)	732 (30.8)	617 (26.0)	296 (12.5)

*Note.* CAS: Castilian Language and Literature; HES: History of Spain; ING: English Language; MAT: Mathematics; MCS: Applied Mathematics for the Social Sciences; VAL: Valencian Language and Literature.

**Table 3:** Severity of each board for the participating universities.

			Unweighted Fit			Weighted Fit		
Trial	Severity	Standard Error	MNSQ	CI	T	MNSQ	CI	T
1-1	0.253	0.054	1.05	(0.62–1.38)	0.3	1.02	(0.59–1.41)	0.1
1-2	−0.179	0.055	0.92	(0.60–1.40)	−0.3	0.93	(0.58–1.42)	−0.3
1-3	0.476	0.052	1.13	(0.64–1.36)	0.7	1.13	(0.62–1.38)	0.6
1-4	0.032	0.052	1.01	(0.61–1.39)	0.1	1.04	(0.59–1.41)	0.2
1-5	−0.345	0.053	1.04	(0.61–1.39)	0.3	0.99	(0.59–1.41)	−0.1
1-6	0.478	0.055	0.95	(0.60–1.40)	−0.2	0.92	(0.59–1.41)	−0.4
1-7	0.373	0.057	1.08	(0.59–1.41)	0.4	0.98	(0.55–1.45)	0.0
1-8	0.459	0.052	1.02	(0.60–1.40)	0.2	0.99	(0.58–1.42)	0.0
1-9	0.555	0.053	0.97	(0.63–1.37)	−0.1	0.99	(0.60–1.40)	−0.1
1-10	0.624	0.061	0.92	(0.47–1.53)	−0.2	0.91	(0.44–1.54)	−0.3
1-11	−0.944	0.059	1.11	(0.54–1.46)	0.5	1.09	(0.52–1.48)	0.3
2-1	−0.576	0.054	1.03	(0.62–1.38)	0.2	1.07	(0.60–1.40)	0.3
2-2	0.363	0.058	1.03	(0.52–1.48)	0.2	1.06	(0.51–1.49)	0.2
2-3	−0.187	0.06	1.01	(0.54–1.46)	0.1	1.02	(0.48–1.52)	0.0
2-4	−0.102	0.059	0.99	(0.55–1.45)	0.1	0.98	(0.53–1.47)	−0.1
2-5	−0.593	0.055	1.14	(0.61–1.39)	0.7	1.06	(0.58–1.42)	0.3
2-6	0.005	0.054	0.99	(0.63–1.37)	0.0	0.99	(0.59–1.41)	0.0
2-7	−0.313	0.053	1.07	(0.65–1.35)	0.4	0.98	(0.63–1.37)	−0.1
2-8	0.163	0.052	0.98	(0.65–1.35)	−0.1	0.93	(0.63–1.37)	−0.4
2-9	−0.418	0.054	1.04	(0.64–1.37)	0.3	1.05	(0.61–1.39)	0.2
2-10	0.047	0.053	1.07	(0.64–1.36)	0.4	1.10	(0.62–1.38)	0.5
3-1	−0.472	0.055	1.13	(0.50–1.40)	0.7	1.09	(0.58–1.42)	0.5
3-2	0.244	0.052	0.99	(0.64–1.36)	0.0	0.96	(0.62–1.38)	−0.2
3-3	−0.089	0.055	0.84	(0.61–1.39)	−0.8	0.87	(0.58–1.42)	−0.7
3-4	−0.178	0.050	0.88	(0.66–1.34)	−0.6	0.87	(0.65–1.35)	−0.7
3-5	0.126	0.056	1.16	(0.62–1.38)	0.9	1.11	(0.59–1.41)	0.6
3-6	0.068	0.050	1.08	(0.67–1.33)	0.5	1.06	(0.64–1.36)	0.4
3-7	−0.235	0.048	1.13	(0.68–1.32	0.8	1.10	(0.66–1.34)	0.6
3-8	0.153	0.052	1.10	(0.63–1.37)	0.6	1.06	(0.60–1.40)	0.2
3-9	−0.537	0.050	1.04	(0.67–1.33)	0.3	1.04	(0.65–1.35)	0.2
3-10	−0.049	0.053	0.95	(0.63–1.37)	−0.2	0.89	(0.61–1.39)	−0.5
3-11	0.276	0.051	0.95	(0.65–1.35)	−0.2	0.95	(0.64–1.36)	−0.2
3-12	0.187	0.053	1.06	(0.62–1.38)	0.4	0.96	(0.59–1.41)	−0.2
3-13	−0.185	0.054	0.94	(0.64–1.36)	−0.3	0.97	(0.62–1.38)	−0.1
3-14	0.661	0.056	1.03	(0.60–1.40)	0.2	1.00	(0.58–1.42)	0.0
3-15	−0.107	0.054	1.04	(0.63–1.37)	0.3	1.03	(0.62–1.38)	0.2
4-1	0.035	0.052	1.16	(0.65–1.35)	0.9	1.11	(0.62–1.38)	0.6
4-2	0.093	0.052	0.98	(0.64–1.36)	0.0	0.93	(0.61–1.39)	−0.3
4-3	0.307	0.050	1.08	(0.65–1.35)	0.5	1.07	(0.63–1.37)	0.4
4-4	−0.386	0.054	1.08	(0.65–1.35)	0.5	1.05	(0.61–1.39)	0.3
4-5	0.316	0.050	1.00	(0.69–1.31)	0.1	0.94	(0.66–1.34)	−0.3
4-6	−0.127	0.052	1.05	(0.67–1.33)	0.4	1.03	(0.64–1.36)	0.2
4-7	−0.121	0.053	1.16	(0.66–1.34)	1.0	1.08	(0.62–1.38)	0.4
4-8	0.658	0.050	1.16	(0.65–1.35)	0.9	1.12	(0.61–1.39)	0.5
4-9	0.074	0.054	1.04	(0.63–1.37)	0.3	0.96	(0.61–1.39)	−0.2
4-10	−0.156	0.057	0.99	(0.58–1.42)	0.0	0.97	(0.54–1.46)	−0.1
4-11	0.698	0.048	1.05	(0.66–1.34)	0.3	1.02	(0.65–1.35)	0.1
4-12	−0.197	0.053	1.01	(0.64–1.36)	0.1	0.97	(0.61–1.39)	−0.2
5-1	0.085	0.049	1.06	(0.69–1.31)	0.4	1.00	(0.65–1.35)	0.0
5-2	−0.427	0.053	1.01	(0.62–1.38)	0.1	1.00	(0.60–1.40)	0.1
5-3	0.725	0.055	1.05	(0.58–1.42)	0.3	0.98	(0.56–1.44)	−0.1
5-4	−1.551	0.053	1.11	(0.63–1.37)	0.6	1.04	(0.61–1.39)	0.2
5-5	0.161	0.053	0.96	(0.60–1.40)	−0.1	0.91	(0.58–1.42)	−0.4
5-6	−0.222	0.389	1.05	(0.63–1.37)	0.3	1.01	(0.60–1.40)	0.0

*Note*: 1-1 to 1-11: University of Alicante; 2-1 to 2-10: University Miguel Hernández; 3-1 to 3-15: University of Valencia; 4-1 to 4-12: Polytechnic University of Valencia; 5-1 to 5-6: University Jaume I; MNSQ: mean square; CI: Confidence Interval; T: Wilson–Hilferty transformation.

**Table 4:** The severity of the boards for each subject.

			Unweighted Fit			Weighted Fit		
Trial	Severity	Standard Error	MNSQ	CI	T	MNSQ	CI	T
CAS	−0.099	0.021	0.94	(0.95–1.05)	−2.3	0.95	(0.95–1.05)	−1.8
HES	−1.070	0.022	0.98	(0.95–1.05)	−0.6	1.01	(0.95–1.05)	0.4
ING	−0.675	0.021	1.07	(0.95–1.05)	2.4	1.07	(0.95–1.05)	2.6
MAT	−0.015	0.024	0.96	(0.93–1.07)	−1.1	0.99	(0.91–1.09)	−0.2
MCS	0.611	0.024	1.20	(0.91–1.09)	4.2	1.19	(0.92–1.08)	4.3
VAL	−0.736	0.097	0.95	(0.94–1.06)	−1.7	0.96	(0.94–1.06)	−1.3

*Note.* CAS: Castilian Language and Literature; HES: History of Spain; ING: English Language; MAT: Mathematics; MCS: Applied Mathematics for the Social Sciences; VAL: Valencian Language and Literature; MNSQ: mean square; CI: Confidential Interval; T: Wilson–Hilferty transformation.

**Table 5:** Mean and standard deviation of severity in subjects for each university.

	(1)	(2)	(3)	(4)	(5)
Subject	Mean (SD)	Mean (SD)	Mean (SD)	Mean (SD)	Mean (SD)
CAS	−0.154 (0.387)	−0.075 (0.436)	−0.052 (0.462)	0.224 (0.395)	0.078 (0.704)
HES	−0.061 (0.402)	−0.063 (0.578)	0.072 (0.848)	−0.037 (0.595)	0.145 (0.569)
ING	0.046 (0.602)	0.008 (0.311)	−0.104 (0.412)	−0.041 (0.332)	0.135 (0.824)
MAT	0.064 (0.710)	0.055 (0.259)	0.032 (0.428)	−0.153 (0.409)	0.017 (0.800)
MCS	0.037 (0.700)	0.063 (0.609)	0.082 (0.377)	−0.201 (0.501)	0.023 (0.587)
VAL	0.072 (0.937)	0.734 (2.180)	0.022 (0.435)	0.266 (0.586)	0.504 (0.551)

*Note.* CAS: Castilian Language and Literature; HES: History of Spain; ING: English Language; MAT: Mathematics; MCS: Applied Mathematics for the Social Sciences; VAL: Valencian Language and Literature; (1) University of Alicante; (2) University Miguel-Hernández of Elche; (3) University of Valencia; (4) Polytechnic University of Valencia; (5) University Jaume I of Castellon.

## Data Availability

Raw data cannot be provided by the authors due to private policy from the Regulation Service of the Valencian Community.
